# Supplementary material for: Do count-based differential expression methods perform poorly when genes are expressed in only one condition?
Source: Genome Biol. 2015 Oct 8;16:222. doi: 10.1186/s13059-015-0781-3 (PMC4599205; doi:10.1186/s13059-015-0781-3)
Supplement: Additional file 2 — Supplementary figures. This file contains the mentioned supplementary figures. (PDF 712 kb) [file 13059_2015_781_MOESM2_ESM.pdf]

Supplementary Figure for "Do count-based differential expression methods perform poorly when genes are expressed in only one condition?"

Xiaobei Zhou and Mark D. Robinson

August 20, 2015

List of Supplementary Figures

1 (correct) normalization factors used in edgeR 1
2 Relationship among S/N, mean, variance and dispersion for the ENCODE dataset . 2
3 mean-variance relationships for S/N calculated on different scales and their corresponding ROC curves 3
4 Comparison of the frequency of all-zeros occuring in real RNA-seq datasets and simulation 4
5 Comparison of dispersion estimates before and after introduction of zeros 5

1 Supplementary Figure

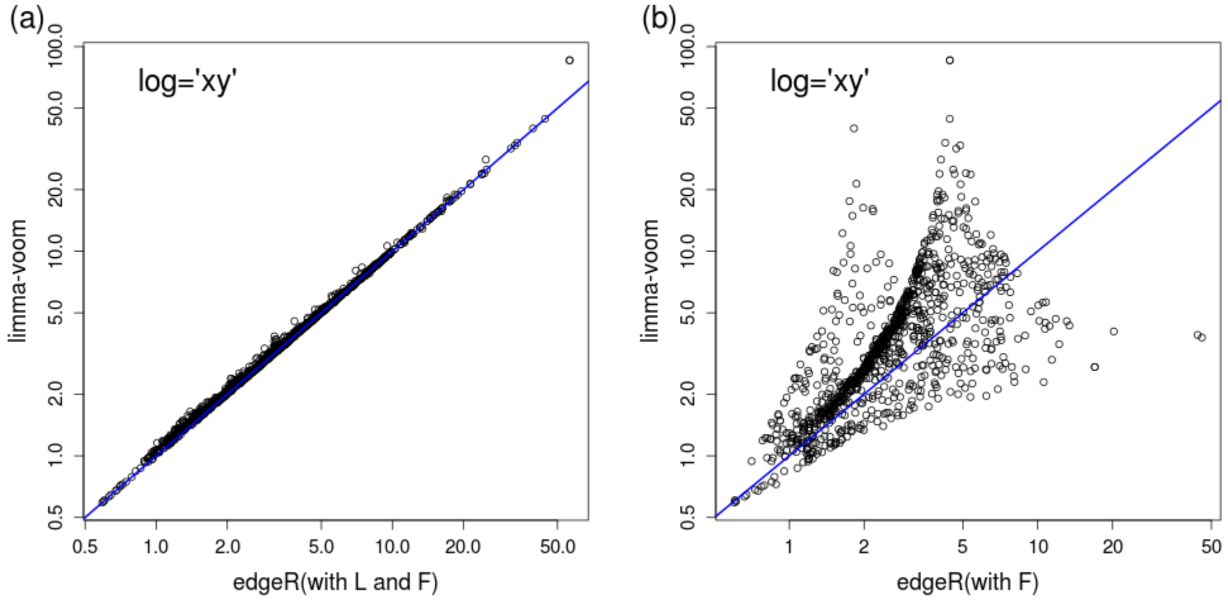

Supplementary Figure 1: Normalization factors used in edgeR. (a) Here, scatter plots of S/N (signal-to-noise) between limma-voom and edgeR are shown, here correctly using both the library size and normalization factors. (b) As shown in Figure 1 of the main manuscript, scatter plot of S/N (signal-to-noise) between limma-voom and edgeR are shown, incorrectly using only the normalization factors (see code in Additional file 3); in edgeR, both factors should be multiplied together. In both cases, the comparison made is between GM12892 cells and H1-hESC cells.

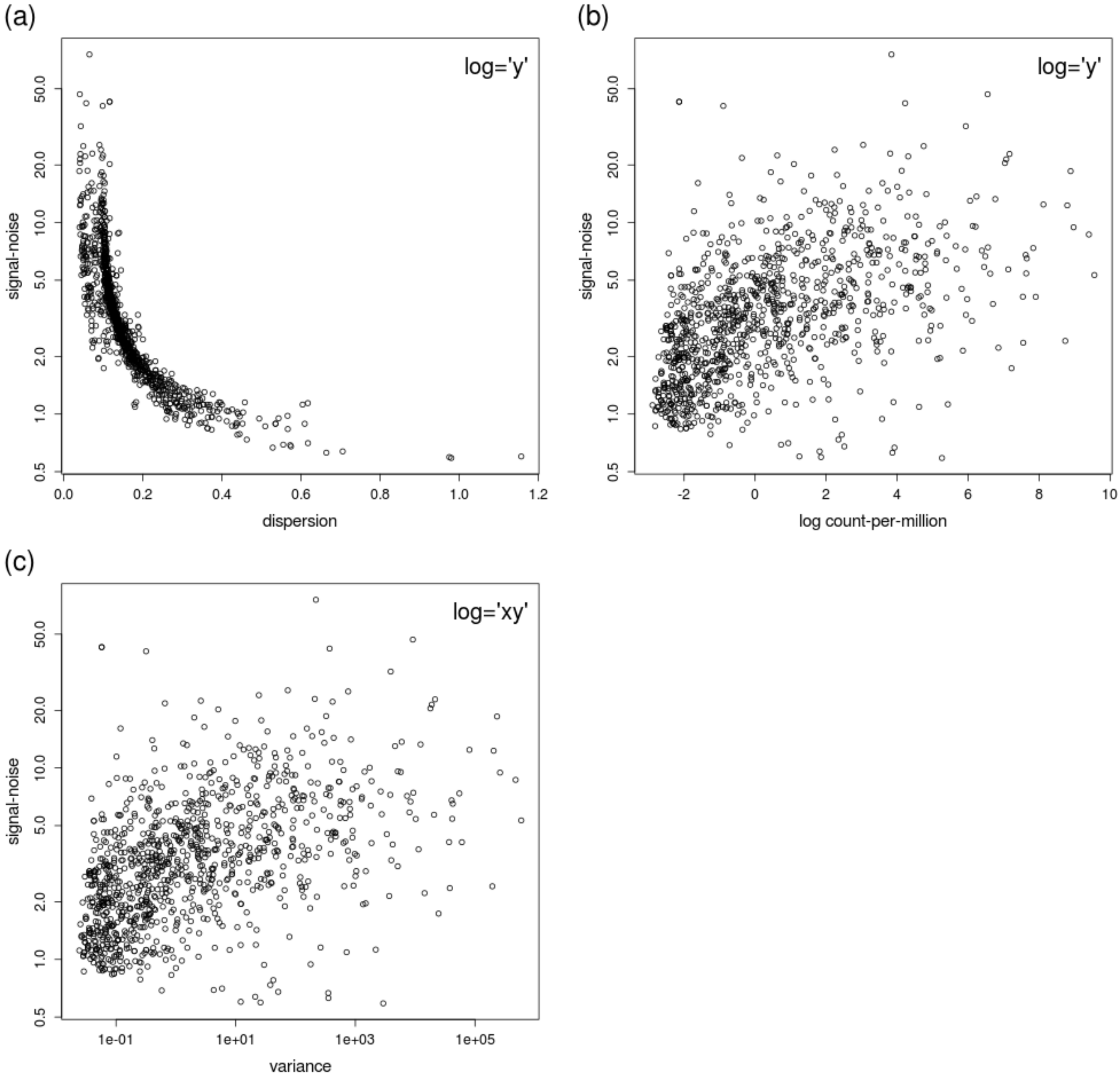

Supplementary Figure 2: Relationship among S/N, mean, variance and dispersion of the expressed-in-one-condition for the ENCODE data (GM12892 versus H1-hESC). (a) S/N versus dispersion. (b) S/N versus log count-per-million. (c) S/N versus estimated variance; since the variability is low for this dataset, panels (b) and (c) look similar.

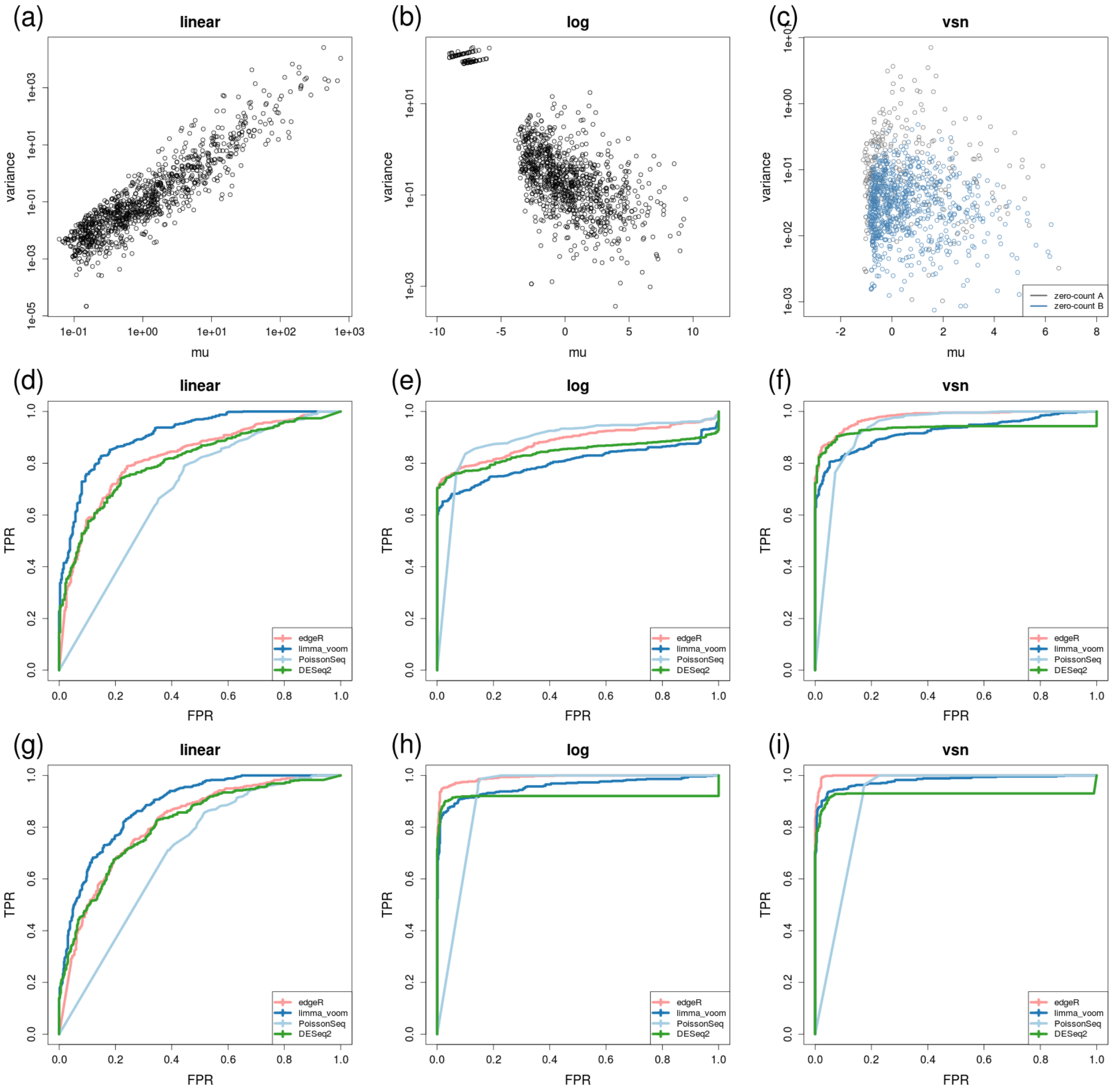

Supplementary Figure 3: Panels (a)-(c) give mean-variance relationships for different scales of the original all-zero-in-one-condition data. Panels (d)-(f) give corresponding ROC curves for the ENCODE dataset (GM12892 cells to H1-hESC), using S/N to set the true labels. ROC curves employ an common truth: the cutoff of S/N; the top 30% as true differentially expressed (DE) and the lowest 30 % as non-DE.Panels (h)-(i) are similar with panels (d)-(f) with a cutoff of S/N: the top 50% as true differentially expressed (DE) and the lowest 50 % as non-DE.

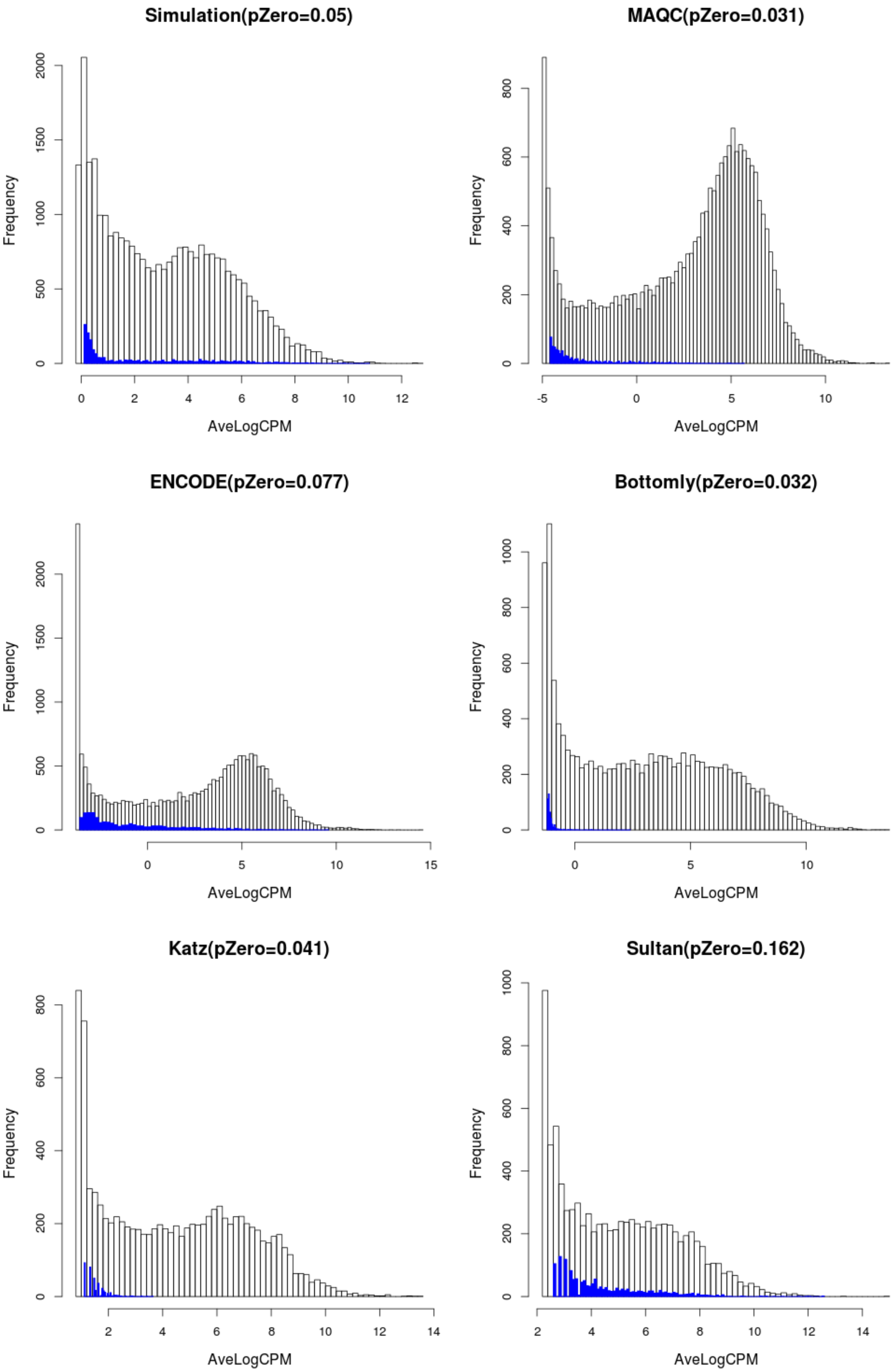

Supplementary Figure 4: Comparison of the frequency of all-zeros that occur in real RNA-seq datasets and simulation. The simulation contains 5% zero-count-in-one-condition. MAQC and ENCODE datasets are those used in the original paper, plus another 3 from Recount project [?].

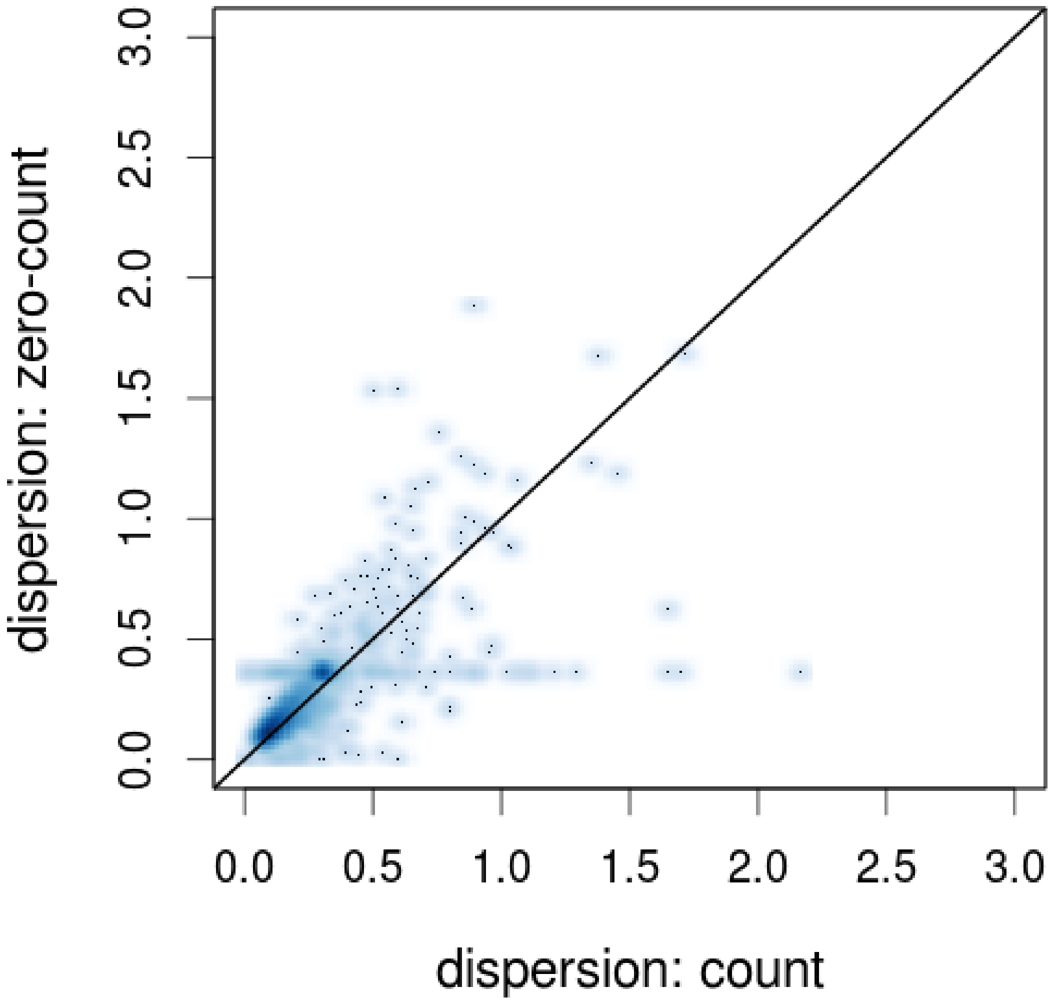

Supplementary Figure 5: This plot shows the dispersion estimates calculated from the single non-zero condition (i.e., estimation after excluding the all-zero condition) to the original non-zero-in-both conditions from simulation data. Only points with zero-counts introduced into 1 condition are shown.
